# Editorial: Public health for chronic kidney disease prevention and care

**DOI:** 10.3389/fpubh.2022.1021075

**Published:** 2022-09-13

**Authors:** Ming-Yen Lin, Marco Fiorentino, I-Wen Wu

**Affiliations:** ^1^Division of Nephrology, Department of Internal Medicine, Kaohsiung Medical University Hospital, Kaohsiung Medical University, Kaohsiung, Taiwan; ^2^Department of Emergency and Organ Transplantation, Nephrology, Dialysis and Transplantation Unit, University of Bari “Aldo Moro”, Bari, Italy; ^3^Department of Nephrology, Keelung Chang Gung Memorial Hospital Keelung, Keelung, Taiwan

**Keywords:** chronic kidney disease, risk factor, screening, prognosis, precision science

More than 850 million people worldwide suffer from kidney disease (1), but not everyone is fortunate to receive professional medical care and treatments. According to the Global Burden Disease study, chronic kidney disease (CKD) and its associated cardiovascular disease caused 2.6 million deaths and 35.8 million disability-adjusted life losses in 2017, especially in medium or low socio-demographic countries (2). Public health research is vital to explore etiology, disease prevention, early detection, intervention effect evaluation, and reasonable resource allocations. However, it is evident that the mainstream CKD studies in the last decade mainly focused on the assessment of drug treatment effects and the correlation between biomarkers and prognosis, with a lack of studies focusing on the etiology or factors associated with CKD progression in one community. Identifying specific etiology and mechanisms affecting CKD development and progression is the leverage point for arranging subsequent actions (3–5) to prevent and manage CKD.

In this Research Topic, we invite global experts to submit their latest research in an attempt to clarify the public health questions currently facing CKD and to explore possible solutions. Although it is unrealistic to expect such a short-term Research Topic to bring many significant impacts, we hope that it will initiate collaboration and more research focus.

This research involves four accepted articles and summarizes their main contributions and relevant implications below.

## Environmental determinants

Environmental factors can affect CKD development and progression. Individual risk for CKD development and prognosis may be determined by insurance systems, neighborhoods, and occupational environments (6). Wu et al. observed that patients with stage 3b to 5 CKD are exposed long-term to air pollutants [particles measuring <2.5 μm in diameter, PM2.5 level ≥ 31.44 μg/m^3^ or nitrogen dioxide (NO2) ≥ 15.00 ppb] may be associated with higher risks of first-hitting renal function deterioration. It is worth noting that both ranges are more elevated than WHO Air Quality Guidelines (15 μg/m^3^ and 13.3 ppb, respectively) (7). This highlights that governments need to pay more attention to health equity, especially regarding environmental threats to kidney health.

## Progression risk in the community

Although there are varying scales of CKD screening in different countries, timely identification of the risk of CKD progression in a community is challenging. Chu et al.'s study defined overweight as a body mass index ≥ 24 kg/m^2^ and metabolically unhealthy as a Homeostasis Model Assessment of Insulin resistance score ≥ 2.5 in one community cohort. They discovered that metabolically unhealthy people, regardless of body weight, compared to those with metabolically healthy normal weight, increased the incident intensity to a 15% decline in the estimated glomerular filtration rate (eGFR) in the elderly, indicating that other metabolic disorders in addition to body weight should be considered to intervene to prevent renal function decline. Tsai et al. used 10-year screening survey data to establish CKD state transition risk functions by common screening items to improve existing CKD screening practices. It is anticipated that these study findings could inspire more longitudinal community-based studies to offer more evidence based on the natural course of CKD.

## Mortality

The mortality risk, especially caused by cardiovascular disease, is higher than the dialysis risk in the early CKD phase. Vascular calcification may be an early sign of CKD prognosis. Wang et al. surveyed cardiovascular events, including non-fatal myocardial infarction, unstable angina, cerebrovascular events (intraparenchymal hemorrhage, subarachnoid hemorrhage, cerebral infarction), hospitalization for congestive heart failure, serious cardiac arrhythmia (resuscitated cardiac arrest, ventricular fibrillation, sustained ventricular tachycardia, paroxysmal ventricular tachycardia, an initial episode of atrial fibrillation or flutter, severe bradycardia or heart block) and peripheral arterial disease in patients with CKD stages 1–4. Their study found that cardiac valvular calcifications were only associated with cardiovascular events in patients with an eGFR ≥45 ml/min/1.73 m^2^. However, abdominal aortic calcifications were associated with an increased risk of all-cause mortality; thus, more research is needed to explore the roles of valvular calcification sites and their clinical significance in CKD.

In conclusion, CKD is a global public health matter. This research article does not attempt to incur all relevant research in such a short period, but it is the start of a continuous process to bring more evidence, attention, and actions on CKD prevention and care.

## Author contributions

All authors listed have made a substantial, direct, and intellectual contribution to the work and approved it for publication.

## Conflict of interest

The authors declare that the research was conducted in the absence of any commercial or financial relationships that could be construed as a potential conflict of interest.

## Publisher's note

All claims expressed in this article are solely those of the authors and do not necessarily represent those of their affiliated organizations, or those of the publisher, the editors and the reviewers. Any product that may be evaluated in this article, or claim that may be made by its manufacturer, is not guaranteed or endorsed by the publisher.
